# Validity and reproducibility of the Prime Diet Quality Score (PDQS) against a four-day food diary in adults at risk of cardiovascular disease on the island of Ireland

**DOI:** 10.1017/jns.2024.23

**Published:** 2024-08-02

**Authors:** Sarah F. Brennan, Rebecca Finlay, Marina Ferrari, Chris R. Cardwell, Lorraine Brennan, Jayne V. Woodside

**Affiliations:** 1 Centre for Public Health, Institute for Global Food Security, Institute of Clinical Sciences A, Queen’s University Belfast, Belfast, UK; 2 UCD School of Agriculture and Food Science, UCD Institute of Food and Health, Conway Institute, University College Dublin, Dublin, Ireland

**Keywords:** Diet quality, Diet quality scores, Diet score validation

## Abstract

There is an increasing need for valid, rapid diet screening tools. A significant association between the Prime Diet Quality Score (PDQS) and reduced risk of cardiovascular disease (CVD) has been demonstrated in the US but evidence of its use in Europe is lacking. The aim of this study was to amend the PDQS for a UK/Irish population and determine validity and reliability in those at risk of CVD. Participants were recruited via online adverts across the island of Ireland. The PDQS was amended for a UK/Irish population and participants completed PDQS and reference measure (4-day food diary (FD)) on two occasions. PDQS score was calculated directly from PDQS and indirectly from FDs. Validity was determined using Spearman correlation coefficients (SCCs) (*r*), intraclass correlation coefficients (ICCs) and weighted kappa. Reliability was determined using SCCs (*r*), ICCs, weighted kappa and coefficient of variation.

‘Data were available for n = 115 (Month 0) and n = 108 (Month 3) participants for validity and n = 110 for reliability assessment (PDQS completed at both timepoints)’. PDQS score from PDQS was significantly correlated with PDQS score from FDs at months 0 (*r* = 0.59, *P* < 0.01) and 3 (*r* = 0.65, *P* < 0.01), with similar associations observed via ICCs. Weighted kappa indicated moderate agreement. PDQS score at month 0 was significantly correlated with PDQS score at month 3 (*r* = 0.78, *P* < 0.01), with similar associations observed via ICCs. Weighted kappa indicated moderate agreement. Results indicate that the amended PDQS is a valid and reliable tool to determine diet quality in a UK/Irish population at risk of CVD.

## Introduction

It is thought that up to 80% of cardiovascular diseases (CVD) and over one-third of cancers could be prevented by modifying behaviours such as diet, smoking, alcohol consumption and physical activity.^(1)^ Despite this, the global burden of non-communicable diseases (NCDs) has continued to rise in recent decades, with 71% of worldwide deaths attributable to NCDs.^(1)^

A healthy dietary pattern is known to reduce the risk of NCDs.^(2–6)^ However, accurate assessment of dietary intake is vital to determine how best to identify those with suboptimal dietary intake. Commonly utilised methods of dietary assessment, including food frequency questionnaires (FFQs), 24 h recalls and food diaries (FD), are burdensome to both research participants and researchers^(7)^ and the American Heart Association has called for widespread adoption of valid, rapid diet screener tools in primary care and relevant prevention settings to help easily identify suboptimal dietary intake and reduce incidence and improve management of NCDs.^(8)^

A systematic review of brief (<35 items) dietary questionnaires suitable for clinical use in the prevention and management of obesity, CVD and type 2 diabetes was published in 2015 and reported on 35 tools, 20 of which had been developed for use in the United States (US).^(9)^ Authors concluded that the tools evaluated are suitable for guiding clinicians but must be adapted and evaluated locally to ensure acceptable levels of relative validity and reliability for the population under study.^(9)^

Since the publication of this systematic review, several other US studies have developed/amended such tools.^(10–12)^ In a study that prospectively examined the association between diet quality assessed using three different diet quality indexes and CVD risk in three US cohorts, the 21-item Prime Diet Quality Score (PDQS), which included both ‘healthy’ and ‘unhealthy’ food groups, was inversely associated with CVD risk in all three cohorts. In contrast, the Food Group Index and the Minimal Diet Diversity Score for Women, which only accounted for ‘healthy’ food groups, had only a limited association with CVD risk across the cohorts.^(11)^ It has been suggested that the PDQS has a simpler scoring system than other diet quality scores despite its greater gradation of scoring, which enables rapid administration and better categorisation of diet quality. Furthermore, it was better able to predict CVD than the other scores in both young and older men and women.^(11)^ As such, it has been suggested that a tool such as the PDQS could be utilised as an efficient field tool to screen diet quality in place of more burdensome dietary assessment methods.^(11)^ Several more recent studies have adapted the PDQS, firstly adapting a 22-item PDQS to be administered either as a 24-hour recall or with a 30-day reference period,^(13)^ and secondly developing a rapid 13-item rPDQS^(14)^; each of these has been validated against food group equivalents and Healthy Eating Index-2015 (HEI-2015) scores estimated from Automated Self-Administered 24-hour (ASA24) Dietary Assessment Tools in US populations.^(13,14)^ These versions of the PDQS have also been associated with several NCD outcomes including coronary artery disease, all-cause mortality and hypertension in pregnancy and gestational diabetes.^(11,15–18)^ The Global Diet Quality Score (GDQS), a further diet screening tool which was also based on the PDQS, has also been associated with NCD-related outcomes in nonpregnant, nonlactating US women of reproductive age.^(19)^ These studies have therefore highlighted the broad applicability of the PDQS in terms of its association with various non-communicable disease health outcomes.

Research into CVD-specific diet screening tools in European populations is lacking. Various adaptations of the PDQS have demonstrated its ability to predict CVD risk in multiple population groups. It has been suggested that future studies should investigate if this efficient field tool is associated with a wider range of health outcomes relevant to diverse populations in both high- and low-income countries,^(11)^ so it is of interest to adapt and test this tool for a European population, in order to account for regional differences in dietary patterns and food preparation habits, prior to use in future research. This study aimed to adapt the original PDQS dietary screening tool for a UK/Irish population and determine its validity and reliability against a 4-day FD in a population at risk of CVD.’

## Methods

This was a dual-centre study completed at both Queen’s University Belfast (QUB), Northern Ireland and University College Dublin (UCD), Republic of Ireland. This study was conducted according to the guidelines laid down in the Declaration of Helsinki and all procedures involving human subjects/patients were approved by the both the Faculty of Medicine, Life and Health Sciences QUB (Reference number: MHLA 21_92) and the UCD Sciences Human Research Ethics Committee (Reference number: LS-20-02-Brennan).

### Amendment of PDQS for population on the island of Ireland

The US version of the PDQS is a 21-item dietary screening tool previously described by Fung *et al.* ^(11)^ and based on the PrimeScreen.^(10)^ Food groups are classified as ‘healthy’ or ‘unhealthy’ based on the scientific evidence with regards to their direction of association with the risk of NCDs, and their nutrient contribution across various global regions.^(11)^ The food groups considered ‘healthy’ in the original PDQS dietary screening tool were: dark green leafy vegetables, cruciferous vegetables, carrots, other vegetables, whole citrus fruits, other whole fruits, legumes, nuts, poultry, fish, eggs, whole grains, and liquid vegetable oils. The food groups considered ‘unhealthy’ were: red meat, potatoes, processed meat, whole milk dairy, refined grains, baked goods, sugar-sweetened beverages, fried foods obtained away from home, desserts and ice cream. Points are assigned according to the following criteria for ‘healthy’ food groups: 0–1 serving per week (0 point), 2–3 servings per week (1 point) and ≥4 servings per week (2 points) and scoring is reversed for ‘unhealthy’ food groups.

In order to amend the original PDQS dietary screening tool for a UK/Irish population, researchers based at both sites reviewed original PDQS food groups and amended these, where appropriate, to reflect any differences in consumption habits or dietary recommendations between the US and UK/Ireland regions.^(20,21)^ All decisions regarding amendments were reached by consensus by the research team who are all research nutritionists or dietitians, are based in the UK and Ireland and have extensive experience in utilisation and review of dietary assessment tools for various population groups in these regions.

The amendments are as follows: the food category ‘potatoes’ were categorised as ‘unhealthy’ in the original version of the PDQS but were re-classified as ‘healthy’ in the amended PDQS with accompanying examples listed as ‘boiled, baked, mashed’ rather than ‘chips/fries or roast potatoes’ as listed in the original PDQS to reflect UK/Ireland cooking methods. Unhealthy potato products such as chips, roast potatoes and crisps listed in the original PDQS under the ‘potatoes’ group were then incorporated into the ‘high-fat foods’ group examples within the amended PDQS. The categories ‘whole eggs’ and ‘nuts’ were classified as ‘healthy’ within the original US PDQS, but the highest consumption frequency option of ‘twice or more per day’ was given 0 points rather than 2 within amended PDQS, so that the frequency of ‘nearly daily or daily category’ received the highest number of points. This was because consuming nuts or eggs twice or more per day was considered excessive according to current regional dietary recommendations.^(20,21)^ The amended PDQS dietary screening tool utilised in this validation and reliability assessment is presented in Table 1 with colour coding to indicate scoring guidance and will be referred to as PDQS from this point forward.

**Table 1.** Amended Prime Diet Quality Score Dietary Screening Tool for UK/Irish Population

Prime Diet Quality Score						
For each question, mark the column indicating how often on average you have used the item(s) during the past 3 months		Less than once per week	Once per week	2–4 per week	Nearly daily or daily	Twice or more per day
1	Red Meat e.g. minced beef (lasagne, bolognaise, Cottage Pie), beef stew/casserole, steak, pork chop, lamb.	**□**	**□**	**□**	□	□
2	Processed Meats e.g. sausages, bacon, ham, corned beef, tinned meat.	**□**	**□**	**□**	□	□
3	Poultry e.g. chicken or turkey breast/fillet, slices (no batter/crumbs).	□	□	**□**	**□**	**□**
4	Fish e.g. cod, haddock, salmon, tuna, mackerel (no batter/crumbs).	□	□	**□**	**□**	**□**
5	Whole Eggs e.g. boiled, scrambled, poached (not fried).	□	□	**□**	□	□
6	Whole Milk Dairy Foods e.g. whole/full-fat milk, hard cheese, butter, full-fat yoghurts.	**□**	**□**	**□**	□	□
7	High-Fat Foods e.g. fast food takeaways (chips, fried chicken/fish/burgers), fried breakfasts (e.g. fried breads, eggs), roast potatoes, crisps.	**□**	**□**	**□**	□	□
8	Whole-Grain Foods e.g. wholegrain breads, wholegrain breakfast cereals (porridge, Weetabix, Shredded Wheat), brown pasta/rice.	□	□	**□**	□	□
9	Sweet Baked Foods e.g. cakes, buns, muffins, cookies, scones.	**□**	**□**	**□**	□	□
10	Potatoes e.g. boiled, baked, mashed (not chips, roast).	□	□	**□**	□	□
11	Dark Green Leafy Vegetables e.g. spinach, lettuce, kale, spring greens (includes frozen, tinned).	□	□	**□**	□	□
12	Cruciferous Vegetables e.g. broccoli, cauliflower, cabbage, brussels sprouts (includes frozen, tinned).	□	□	**□**	□	□
13	Carrots e.g. raw, boiled, steamed, mashed, microwaved, frozen, tinned.	□	□	**□**	□	□
14	Other vegetables e.g. mushrooms, corn, turnip, cucumber, tomatoes (includes frozen, tinned).	□	□	**□**	□	□
15	Legumes e.g. peas, baked beans, kidney beans, lentils, chickpeas (includes frozen, tinned).	□	□	**□**	**□**	**□**
16	Whole Citrus Fruit e.g. oranges, grapefruit, lemons (not fruit juices).	□	□	**□**	□	□
17	Other fruits e.g. apples, pears, bananas, strawberries, raspberries, grapes, melon (includes frozen, tinned).	□	□	**□**	□	□
18	Liquid Vegetable Oils e.g. olive oil, rapeseed oil (not palm oil or coconut oil).	□	□	**□**	**□**	□
19	Nuts e.g. almonds, peanuts, cashew, hazelnuts, brazil nuts (unsalted only).	□	□	**□**	**□**	□
20	Desserts, Puddings and Confectionery e.g. ice cream, custard, sponge puddings, crème brulee, fruit pies, cheesecakes, chocolate, sweets.	**□**	**□**	**□**	□	□
21	Sugar Sweetened Beverages e.g. cola, sodas, lemonade, flavoured juices, energy drinks (not diet/sugar-free varieties).	**□**	**□**	**□**	□	□

Scoring system: red = 0, yellow = 1, green = 2. Score range 0–42.

### Study population and recruitment

Study eligibility criteria at both sites were as follows: participants were eligible if they were aged 45 years or over and had one or more of the following risk factors for CVD: current Body Mass Index (BMI) of 25 kg/m^2^ or over; a current smoker, diagnosed hypertension (elevated blood pressure of 140/90 mmHg or above diagnosed by a medical professional) or diagnosed hypercholesterolaemia (elevated total cholesterol of 5 mmol/l or above diagnosed by a medical professional). Those being pharmacologically managed for hypertension or hypercholesterolaemia were also considered at risk of CVD. Participants were excluded if they had a medical condition which required significant dietary management (e.g. diet-only management of type 2 diabetes; active inflammatory bowel disease), dietary restriction(s) that would substantially limit their ability to complete the study requirements (e.g. food allergy, Coeliac Disease), excessive alcohol consumption (>28 units/week for men or >21 units/week for women) or if they were unable to provide informed consent.

Standardised recruitment protocols were implemented across both sites. Study recruitment commenced just before the onset of the COVID-19 pandemic in February 2020 and consequently paused between March and June 2020 during national lockdowns. The original intention of the validation and reliability assessment was to validate the PDQS against a 4-day FD and to assess agreement of the PDQS with urinary dietary biomarker levels. Due to requirement to progress with the research remotely during the pandemic, the study protocol was amended to remove collection of biological samples and analysis of urinary biomarkers and, after ethical amendment approval, recruitment re-commenced remotely in June 2020. Recruitment posters were distributed online via online community notices, social media posts and some workplace email distribution lists. Contact details of the research team were provided on the poster and interested participants made contact with the research team via phone or email. Eligibility was determined via telephone with a study researcher. Participant Information Sheets were distributed to those eligible and researchers answered any queries via telephone. Consent was obtained via Qualtrics online survey software (Qualtrics, Provo, UT).

The study aimed to examine the level of agreement between two methods of measurement, so a sample size of 100 was considered appropriate, giving a 95% CI ± 0.34 SD approximately.^(22)^ This sample size calculation was also supported by a comprehensive dietary questionnaire validation review by Cade *et al.* ^(23)^ which recommended that a sample size of at least 50–100 participants is desirable for FFQ validation.^(23,24)^ The QUB site aimed to recruit n = 70 participants, whilst the UCD site aimed to recruit n = 30 participants.

## Study design

To determine criterion-related validity, the test measure (PDQS) was administered to participants followed immediately by the reference measure (4-day FD) on two occasions (month 0 and month 3) to enable comparison between methods. Reliability was determined by comparing the test measure (PDQS) administered at both timepoints (month 0 and month 3). The 3-month interval time frame was intended to account for any seasonal variations in dietary intake, but a short enough time frame to avoid capturing major changes in dietary habits that are more likely to occur years apart.^(23)^

### Administration of the test and reference measures

The PDQS was administered to participants via online survey software. Participants were asked to complete the PDQS with reference to their dietary intake over the previous 3-month period and researchers discussed the PDQS over the phone with participant prior to administration to ensure as accurate an estimation of dietary intake as possible and offered to assist the participant via telephone during completion if necessary. A 4-day FD was used as the reference measure for assessment of criterion-related validity and was posted to participants at home at months 0 and 3 after completion of the PDQS. Participants were asked to record everything they ate and drank over 4 consecutive days (3 weekdays and 1 weekend day) and to include brand names of foods, cooking methods and whether fat/sauces were added to foods. Participants were asked to weigh foods or use household measures to estimate intake and leftovers and provide ingredients and amounts used in preparing a composite dish. Portion-size information and guidance were given to participants via telephone and portion-size photo guides and advice were included within the FD. Participants were asked to contact the researchers if they had any questions during the recording period and were given a pre-paid envelope to return their completed FD. Completed FDs were reviewed by researchers who followed up with a phone call if further details were necessary.

## Feedback

As this study aimed to adapt the PDQS for a UK/Irish population at risk of CVD, comments and feedback in relation to the format and clarity of the questionnaire were informally collected from participants during the study to indicate whether further amendments were necessary.

## Data management

### Calculating PDQS score from PDQS and food diary data

#### PDQS (test measure)

The PDQS score was calculated directly from PDQS responses completed by participants at month 0 and month 3 using the criteria previously detailed and outlined in Table 1 (score range 0–42).

#### Food diary data (reference measure)

FDs were coded using the Nutritics online dietary survey software (Nutritics. (2019). Research Edition (v5.09) [Computer software]. Dublin). In order to ensure standardisation of data entry across sites, 10% of FDs completed at each site were entered in duplicate, reviewed and compared. Any discrepancies identified between sites in the coding of dietary data were discussed and resolved with the wider research team.

Researchers reviewed food files obtained from Nutritics and coded all PDQS-relevant food items so that they corresponded with the food groups 1–21 listed within the PDQS. A list of more ambiguous food items was identified by researchers at both sites and a coding guide was developed to assist with coding these food items (Supplementary Files Table 1).

Intake of each of the coded food items was quantified by calculating total weight in grams (g) consumed for each of the PDQS coded food items (1–21) over the course of the 4-day FD recording period for each participant at each timepoint, e.g. all food items which were coded within the ‘red meat’ category were collated under PDQS group 1 and total amount in grams was summed. The research team estimated an average portion size for each of the 21 PDQS food groups by listing published portion sizes^(25)^ for each example food listed within each PDQS category and calculating an average portion size for each of the 21 food groups (Supplementary Files Table 2). Guidance regarding portions of food typically consumed in multiple units at a time (e.g. 2 sausages, 2 slices of bread, 2 eggs) was obtained from the British Nutrition Foundation ‘Find Your Balance’ Full Portion Size List.^(26)^ Contributing weights within composite dishes listed as examples within the ‘red meat’, ‘poultry’, ‘fish’ or ‘legumes’ food groups, were capped e.g. contributing weight of red meat within Bolognese was capped at 140 g of minced beef, the published portion size.^(19)^

Total weight (g) consumed for each of the 21 PDQS groups was converted to total servings per day over the 4-day recording period by dividing by the average portion size calculated for each of the 21 food groups as described above and converting to a weekly serving estimate by multiplying by 1.75. The weekly serving estimate was converted to the corresponding PDQS frequency categories as follows: 0–0.49 servings per week corresponded with ‘less than once per week’, 0.5–1.49 servings per week corresponded with ‘once per week’, 1.5–4.49 servings per week corresponded with ‘2–4 per week’, 4.5–13.49 servings per week corresponded with ‘nearly daily or daily’ and 14+ servings per week corresponded with ‘twice or more per day’. This enabled the research team to calculate a PDQS score for each participant at each timepoint.

### Additional outcomes

A short lifestyle questionnaire was administered to participants at month 3 via online survey software to capture any notable changes in lifestyle or health over the 3-month study period.

### Statistical analyses

To compare PDQS score derived from PDQS and PDQS score from food diaries, Wilcoxon Signed Rank Test, Mann-Whitney *U*-Test and Kruskal-Wallis *H* Tests were used.

To determine validity of the PDQS, total PDQS scores and individual PDQS food group scores derived from both the PDQS and the 4-day FD at both timepoints were compared. Spearman correlation coefficients (*r*) and intraclass correlation coefficients were obtained for total PDQS scores and individual PDQS food groups derived from both the test and reference measures. The ability of the PDQS to categorise participants into equal thirds of total PDQS from FD data was assessed by weighted kappa, with values of K > 0.8 considered to indicate almost perfect agreement, 0.61–0.80 substantial agreement, 0.41–0.60 moderate agreement, 0.21–0.40 fair agreement, 0.0–0.20 slight agreement, and 0 poor or disagreement.^(27)^ Statistical analyses were performed using SPSS version 20.0 (SPSS Inc, Chicago, IL).

To determine the reliability of the PDQS, Spearman correlation coefficients (*r*) and intraclass correlation coefficients were obtained for total PDQS score derived from the PDQS test measure at both timepoints. Weighted kappa was performed to determine ability of the PDQS to categorise participants into equal thirds of total PDQS score at month 0 and month 3. Coefficient of variations was also obtained to assess reliability of PDQS administered at month 0 and month 3.

Subgroup analyses were performed by gender. Any dietary intakes from the food diary data which were considered implausible using the <500 kcal and >3500 kcal/d energy intake criteria, as described in previous dietary research,^(28–31)^ were identified and analysed separately, where relevant.

Kruskal-Wallis *H* Tests were used to compare average daily nutrient intake by tertile of PDQS total score from amended PDQS.

Data from the changes in lifestyle questionnaire were limited in terms of changes reported but were analysed in terms of changes reported in frequencies and percentages.

## Results

In total, n = 130 participants were screened for study eligibility across both sites. Of these, n = 120 were considered eligible and were recruited. As per study protocol, n = 10 of the screened participants were ineligible to take part for the following reasons: n = 4 had a dietary restriction that would substantially limit their ability to complete study requirements, n = 3 had been diagnosed with Type 2 diabetes and were either on dietary management and/or medication, n = 1 had a history of a previous cardiac event, n = 1 wasn’t able to fulfil collection of data at Month 3 due to relocation and n = 1 reported excessive alcohol consumption.

**Fig. 1.**
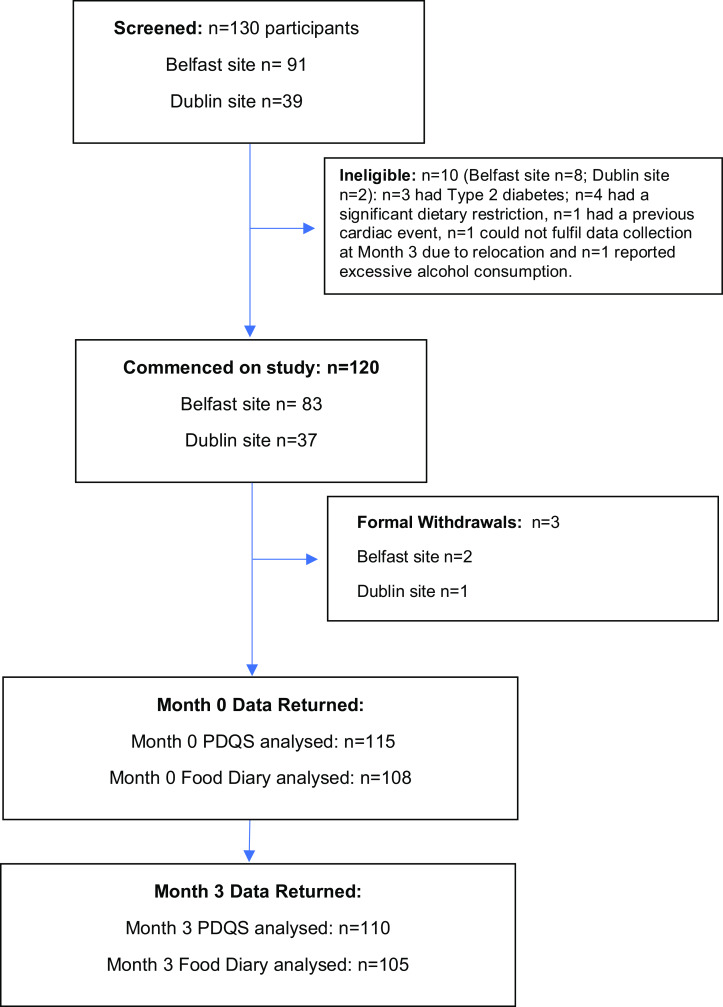
Flow diagram illustrating study recruitment.

Numbers included in validity analysis were the number of participants who completed both the PDQS and the food diary at each timepoint to allow validity assessment, whereas the numbers included in the reliability analysis were the numbers of participants who completed the PDQS at both timepoints to allow reliability assessment. For validity assessment, data were complete for n = 115 participants at month 0 and n = 108 participants at month 3; for reliability assessment, data were complete for n = 110 participants (Fig. 1). No implausible energy intakes were reported according to the <500 kcal and >3500 kcal/d criteria^(28–31)^ so it was not necessary to exclude any participants from the analyses.

**Fig. 2.**
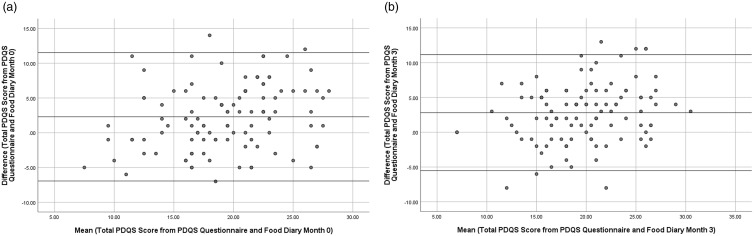
(a) Bland-Altman Plot showing difference between total Prime Diet Quality Score (PDQS) score from PDQS Questionnaire and Food Diary against mean of total PDQS score from PDQS Questionnaire and Food Diary at Month 0 (n = 108). Mean difference: 2.29. (b) Bland-Altman Plot showing difference between total PDQS score from PDQS Questionnaire and Food Diary against mean of total PDQS score from PDQS Questionnaire and Food Diary at Month 3 (n = 105). Mean difference: 2.82.

Sample demographics are presented in Table 2. Mean age of participants was 59.0 years (SD: 9.7), the majority of participants were female (78.3%), 38.3% of participants were classified as overweight and 51.3% with obesity according to BMI (kg/m^2^), 43.5% of participants reported hypertension, 40.9% reported hypercholesterolaemia and 15.7% currently smoked.

**Table 2.** Sample demographics

N = 115	Mean (range)
Age	59.0 (45–89)
BMI (kg/m^2^)	31.2 (18.4–68.8)
	N (%)
Male	25 (21.7)
Female	90 (78.3)
Underweight	1 (0.9)
Normal Weight	11 (9.6)
Overweight	44 (38.3)
Obese	59 (51.3)
Current smoker	18 (15.7)
Hypertension	50 (43.5)
Hypercholesterolaemia	47 (40.9)

Average total PDQS scores derived from both the PDQS and the food diaries at both month 0 and month 3 are presented in Table 3. Wilcoxon Signed Rank Test indicated that average total PDQS score derived from the PDQS was significantly higher, indicating a better diet quality, at both month 0 (20.4; SD: 5.7) and month 3 (21.0; SD: 5.6) than average total PDQS score derived from food diaries at month 0 (18.2; SD: 4.6) and month 3 (18.3; SD: 4.7; *P* < 0.01 at both timepoints). Mann-Whitney *U*-Test indicated that average total PDQS score from the PDQS was significantly higher in females (21.0; SD: 5.6) than in males (18.2; SD 5.7, *P* < 0.05) at month 0; whilst for PDQS score derived from food diaries, females scored higher (19.0; SD: 4.2) than males (16.1; SD: 5.5) at month 3 (*P* < 0.05). Kruskal-Wallis *H* Test indicated that average total PDQS score obtained from the PDQS was significantly lower, indicating a poorer diet quality, in participants with overweight and obesity compared with participants who were underweight/healthy weight at both month 0 (*P* < 0.01) and month 3 (*P* = 0.01). Average PDQS score derived from food diaries was also significantly lower in participants with overweight and obesity compared with participants who were underweight or healthy weight participants at month 0 (*P* < 0.01), but no significant difference was observed between weight categories at month 3.

**Table 3.** Total Prime Diet Quality Score (PDQS) scores obtained from both questionnaire and food diaries at month 0 and month 3

	Total PDQS (PDQS)			Total PDQS (Food Diary)			Difference between total PDQS derived from PDQS and food diaries	
	Month 0 N Mean (SD)	Month 3 N Mean (SD)	Difference between PDQS between M0 and M3 P^1^	Month 0 N Mean (SD)	Month 3 N Mean (SD)	Difference between PDQS from FD between M0–M3 P^1^	Month 0 P^1^	Month 3 P^1^
All sample	115 20.4 (5.7)	110 21.0 (5.6)	0.11	108 18.2 (4.6)	105 18.3 (4.7)	0.62	<0.01	<0.01
Males	25 18.2 (5.7)	24 19.2 (5.5)	0.38	24 17.6 (5.2)	24 16.1 (5.5)	0.16		
Females	90 21.0 (5.6)	86 21.6 (5.5)	0.21	84 18.4 (4.4)	81 19.0 (4.2)	0.16		
P^2^	0.04	0.06		0.36	0.01			
Underweight/normal weight	12 25.4 (3.7)^a^	12 25.6 (3.6)^a^	0.46	11 21.5 (3.2)^a^	11 20.0 (2.9)	0.21		
Overweight	44 21.1 (5.2)^b^	42 21.5 (5.4)^b^	0.30	42 17.4 (4.3)^b^	42 18.6 (4.9)	0.04		
Obese	59 18.8 (5.8)^b^	56 20.0 (5.5)^b^	0.29	55 18.1 (4.8)^b^	52 17.8 (4.8)	0.53		
P^3^	<0.01	0.01		<0.01	0.29			

Superscript letters denote groups that are significantly different from one another. PDQS score range 0–42; higher score indicates better diet quality.

1 Wilcoxon Signed Rank Test.

2 Mann-Whitney *U*-Test.

3 Kruskal-Wallis *H* Test.

Spearman correlation coefficients (*r*), weighted kappa (K) and ICCs for average total PDQS score from PDQS and food diaries at months 0 and 3 are presented in Table 4. Average total PDQS score from PDQS was significantly correlated with average total PDQS score derived from food diaries at month 0 (*r* = 0.59; *P* < 0.01) and month 3 (0.65; *P* < 0.01). Individual food group scores from PDQS were also significantly correlated with those derived from food diary data for 16 of the 21 food groups at month 0 (all *P* < 0.01); and 20 of the 21 food groups at month 3 (*P* < 0.05 for 2 food groups, *P* < 0.01 for 18 food groups). Similar intraclass correlation coefficients were observed between average total PDQS score from the PDQS and average total PDQS score derived from food diaries at month 0 (0.70; 95% CI: 0.49–0.81) and month 3 (0.42; 95% CI: 0.41–0.86). Weighted kappa indicated moderate agreement between the two PDQS scores at month 0 (0.40; se: 0.07) and month 3 (0.42; se: 0.07). Bland-Altman plots (Fig. 2) indicated that, for 95% of participants, average PDQS scores were within the limits of agreement at month 0 (mean difference: 2.3), whereas at month 3, 90.5% of participants were within limits of agreement (mean difference 2.8).

**Table 4.** Spearman correlation coefficients (*r*), weighted kappa (K) (where appropriate) and intraclass correlation coefficients (ICCs) for Prime Diet Quality Score (PDQS) questionnaire and PDQS scores derived from 4-day food diaries at month 0 and month 3; and Spearman correlation coefficients, ICCs and coefficient of variation (where appropriate) for PDQS questionnaire at month 0 and month 3

PDQS Food Groupings	Month 0 (n = 108)		Month 3 (n = 105)		Reliability (n = 110)		
	Validity (*r*) K (se)	ICC (95% CI)	Validity (*r*) K (se)	ICC (95% CI)	Reliability (*r*) K (se)	ICC (95% CI)	Coeff. of variation
Total PDQS score	0.59^**^ 0.40 (0.07)	0.70 (0.49–0.81)	0.65^**^ 0.42 (0.07)	0.73 (0.41–0.86)	0.78^**^ 0.54 (0.04)	0.88 (0.82–0.92)	0.11
Red meat	0.37^**^	0.55 (0.34–0.69)	0.34^**^	0.53 (0.30–0.68)	0.67^**^	0.79 (0.69–0.86)	–
Processed meat	0.30^**^	0.46 (0.22–0.63)	0.21^*^	0.33 (0.02–0.54)	0.56^**^	0.72 (0.59–0.81)	–
Poultry	0.32^**^	0.44 (0.18–0.61)	0.35^**^	0.49 (0.25–0.65)	0.55^**^	0.70 (0.57–0.80)	–
Fish	0.25^**^	0.36 (0.08–0.56)	0.43^**^	0.54 (0.33–0.69)	0.69^**^	0.81 (0.73–0.87)	–
Whole eggs	0.27^**^	0.47 (0.23–0.63)	0.49^**^	0.68 (0.54–0.79)	0.63^**^	0.79 (0.69–0.85)	–
Whole milk dairy foods	0.13	0.24 (–0.09–0.47)	0.36^**^	0.52 (0.30–0.67)	0.42^**^	0.63 (0.46–0.75)	–
High-fat foods	0.17	0.18 (–0.11–0.41)	0.21^**^	0.21 (–0.10–0.44)	0.22^*^	0.42 (0.16–0.61)	–
Wholegrain foods	0.50^**^	0.69 (0.54–0.79)	0.42^**^	0.67 (0.51–0.77)	0.61^**^	0.77 (0.66–0.84)	–
Sweet baked foods	0.30^**^	0.46 (0.20–0.63)	0.34^**^	0.49 (0.25–0.65)	0.49^*^	0.66 (0.51–0.77)	–
Potatoes	0.33^**^	0.48 (0.23–0.65)	0.46^**^	0.62 (0.43–0.74)	0.64^**^	0.78 (0.68–0.85)	–
Dark green leafy vegetables	0.16	0.18 (–0.11–0.40)	0.46^**^	0.46 (0.01–0.69)	0.61^**^	0.76 (0.65–0.84)	–
Cruciferous vegetables	0.30^**^	0.46 (0.20–0.63)	0.20^*^	0.31 (0.02–0.53)	0.64^**^	0.77 (0.67–0.84)	–
Carrots	0.27^**^	0.38 (0.11–0.58)	0.32^**^	0.50 (0.27–0.66)	0.64^**^	0.77 (0.67–0.85)	–
Other vegetables	0.38^**^	0.45 (0.08–0.66)	0.38^**^	0.49 (0.12–0.69)	0.60^**^	0.75 (0.63–0.83)	–
Legumes	0.37^**^	0.51 (0.29–0.67)	0.13	0.19 (–0.19–0.45)	0.55^**^	0.72 (0.59–0.81)	–
Whole citrus fruit	0.64^**^	0.75 (0.64–0.83)	0.52^**^	0.64 (0.43–0.76)	0.71^**^	0.82 (0.74–0.88)	–
Other fruits	0.59^**^	0.77 (0.67–0.84)	0.60^**^	0.77 (0.66–0.85)	0.67^**^	0.82 (0.74–0.88)	–
Liquid vegetable oils	0.18	0.22 (–0.08–0.45)	0.26^**^	0.32 (–0.05–0.55)	0.67^**^	0.81 (0.72–0.87)	–
Nuts	0.38^**^	0.60 (0.41–0.73)	0.48^**^	0.69 (0.54–0.79)	0.69^**^	0.85 (0.78–0.89)	–
Desserts, pudding, confectionery	0.09	0.18 (–0.19–0.43)	0.25^**^	0.42 (0.15–0.61)	0.44^**^	0.59 (0.41–0.72)	–
Sugar sweetened beverages	0.37^**^	0.53 (0.31–0.68)	0.39^**^	0.67 (0.52–0.78)	0.64^**^	0.78 (0.67–0.85)	–

R = Spearman correlation coefficient; K = weighted kappa statistics, SE, standard error; ICC, intraclass correlation coefficient. ICC (two-way mixed; absolute agreement).

* Significant at the 0.05 level (2-tailed).

** Significant at the 0.01 level (2-tailed).

Reliability assessment indicated that average total PDQS scores obtained directly from PDQS at months 0 and 3 were statistically significantly correlated (*r* = 0.78; *P* < 0.01) and similar intraclass correlation coefficients were observed (0.88; 95% CI: 0.82–0.92). Weighted kappa indicated moderate agreement between PDQS score at the different timepoints (0.54; se: 0.04). Coefficient of variation for average total PDQS obtained at both timepoints was 0.11, indicating low variation between the means.

Subgroup analyses were performed by gender (Supplementary Files Table 3 and 4), although 78.3% of the sample were female. Agreement between measures was statistically significant in both females and males at both months 0 and 3; whilst stronger agreement was observed at month 0 in females compared with males and stronger agreement seen in males compared with females at month 3. However, overall validity and reliability results were consistent with primary analysis. As the majority of the sample was classified either as overweight (38.3%) or obese (51.3%), subgroup analyses were not conducted by BMI category.

Average daily nutritional intake from food-only sources, assessed using food diary data, is presented in Table 5 alongside nutritional intake by tertile of total PDQS score from PDQS at month 0 and month 3. Kruskal-Wallis *H* Test was utilised to determine if there were any statistically significant differences between groups. At month 0, average daily fibre (g) and folate (µg) intakes were significantly higher in those with total PDQS score in highest tertile of PDQS score compared with those who scored in the middle and lowest tertiles (both *P* < 0.01). Average daily potassium, thiamine and riboflavin intakes (mg) were significantly higher in those that scored in the highest tertile of PDQS score compared with those in the lowest tertile of PDQS score (*P* < 0.01; 0.03 and 0.02, respectively), whilst average daily iodine (µg), vitamin C (mg) and vitamin D (µg) intakes were significantly higher in those who scored in the middle and highest tertile of PDQS score compared with those who scored in the lowest tertile of PDQS intake (*P* < 0.01; <0.01 and 0.04, respectively).

**Table 5.** Average daily nutrient intake by tertile of Prime Diet Quality Score (PDQS) total score from amended PDQS (food only)

Nutrient	Month 0					Month 3					Difference in nutrient intake Month 0–Month 3 P^**^
		PDQS total score					PDQS total score				
	Average daily intake Mean (sd)	Tertile 1 (n = 36) Mean (sd)	Tertile 2 (n = 38) Mean (sd)	Tertile 3 (n = 41) Mean (sd)	Difference between tertiles P^*^	Average daily intake Mean (sd)	Tertile 1 (n = 31) Mean (sd)	Tertile 2 (n = 40) Mean (sd)	Tertile 3 (n = 39) Mean (sd)	Difference between tertiles P^*^	
Energy (kcal)	1943.1 (492.1)	1944.8 (555.8)	1981.2 (538.8)	1904.6 (384.3)	0.96	1790.3 (490.6)	1886.5 (603.3)	1761.9 (457.8)	1752.8 (438.1)	0.79	<0.01
Carbohydrate (g)	205.1 (52.6)	207.7 (50.7)	210.0 (62.5)	198.2 (43.6)	0.56	189.3 (61.9)	201.3 (81.7)	184.9 (57.6)	185.4 (49.7)	0.76	<0.01
Protein (g)	81.0 (24.0)	77.7 (30.0)	84.0 (21.1)	80.6 (19.8)	0.23	76.6 (19.8)	77.5 (23.2)	74.7 (17.8)	78.0 (19.6)	0.95	0.02
Total Fat (g)	80.2 (26.2)	79.8 (26.1)	82.1 (29.4)	78.9 (23.4)	0.98	73.8 (24.3)	79.2 (24.2)	72.0 (22.6)	71.8 (26.0)	0.40	0.01
Saturated Fat (g)	29.4 (11.4)	30.5 (11.6)	31.4 (13.6)	26.5 (8.0)	0.13	27.8 (10.3)	31.6 (10.8)	27.3 (8.0)	25.7 (11.4)	0.05	0.19
Free Sugars (g)	40.0 (26.1)	44.6 (28.6)	41.6 (30.3)	34.5 (18.1)	0.34	35.8 (23.8)	44.8 (32.4)	34.4 (22.4)	31.0 (15.7)	0.34	0.13
Alcohol (g)	25.3 (17.6)	23.9 (17.4)	25.9 (19.1)	25.8 (17.4)	0.95	20.9 (17.0)	19.9 (17.5)	21.8 (20.0)	20.6 (13.8)	0.84	0.15
NSP Englyst Fibre (g)	15.3 (5.0)	12.7 (4.0)^a^	15.0 (4.9)^a^	17.8 (5.0)^b^	<0.01	14.4 (5.4)	11.6 (5.0)^a^	13.6 (4.3)^a^	17.1 (5.4)^b^	<0.01	0.03
Fibre (g)	20.8 (6.7)	17.4 (5.6)^a^	20.4 (5.7)^b^	24.3 (7.0)^c^	<0.01	19.0 (6.6)	15.4 (5.8)^a^	18.2 (5.5)^b^	22.4 (6.6)^c^	<0.01	<0.01
Iron (mg)	11.6 (3.9)	11.0 (4.4)	11.0 (2.8)	12.6 (4.3)	0.11	11.1 (4.1)	10.5 (4.2)	10.5 (3.2)	12.2 (4.7)	0.12	0.15
Sodium (mg)	2115.6 (687.5)	2139.2 (624.0)	2220.9 (805.3)	1992.5 (610.0)	0.41	2078.3 (758.7)	2210.1 (822.1)	2037.4 (676.9)	2029.2 (800.9)	0.57	0.64
Potassium (mg)	3245.5 (831.6)	2923.0 (923.2)^a^	3252.4 (720.5)^a,b^	3518.7 (766.9)^b^	<0.01	3119.3 (824.7)	2830.7 (799.4)^a^	3023.4 (725.7)^a,b^	3417.3 (861.0)^b^	0.01	0.01
Calcium (mg)	824.9 (258.0)	763.4 (269.2)	864.0 (279.4)	840.3 (220.8)	0.24	781.2 (247.8)	819.7 (225.3)	748.9 (264.7)	788.0 (246.6)	0.29	0.09
Iodine (ug)	145.8 (61.7)	116.3 (35.3)^a^	150.7 (64.6)^b^	166.6 (68.0)^b^	<0.01	144.7 (74.3)	129.8 (67.6)	10.5 (3.2)	12.2 (4.7)	0.12	0.70
Thiamine (mg)	1.6 (0.6)	1.4 (0.6)^a^	1.6 (0.5)^a,b^	1.7 (0.6)^b^	0.03	1.5 (0.5)	1.4 (0.5)	1.4 (0.5)	1.6 (0.5)	0.06	0.02
Riboflavin (mg)	1.7 (0.6)	1.5 (0.6)^a^	1.7 (0.5)^a,b^	1.9 (0.6)^b^	0.02	1.6 (0.6)	1.6 (0.5)	1.6 (0.6)	1.7 (0.6)	0.28	0.01
Niacin (mg)	38.0 (13.2)	38.0 (16.9)	38.7 (11.4)	37.3 (11.5)	0.82	35.9 (0.6)	37.7 (16.3)	35.2 (9.7)	35.3 (11.6)	0.86	0.04
Folate	275.0 (121.0)	230.2 (118.2)^a^	258.7 (82.3)^a^	329.9 (136.1)^b^	<0.01	254.2 (111.0)	227.3 (112.8)^a^	232.5 (69.9)^a^	294.9 (132.8)^b^	<0.01	0.02
Vitamin A (Total Retinol Equivalents) (µg)	843.5 (552.0)	712.9 (403.0)	874.6 (552.9)	926.7 (648.1)	0.40	867.8 (499.5)	714.3 (295.1)^a^	843.2 (437.3)^b^	999.4 (631.3)^b^	0.09	0.76
Vitamin B12 (Cobalamin) (µg)	5.7 (2.8)	5.3 (3.5)	6.0 (2.9)	5.7 (2.1)	0.11	5.1 (2.7)	5.4 (4.1)	4.8 (2.0)	5.2 (2.2)	0.66	0.01
Vitamin C (mg)	95.0 (57.7)	56.8 (32.6)^a^	98.1 (49.7)^b^	125.3 (63.7)^b^	<0.01	84.1 (58.7)	50.6 (39.3)^a^	74.8 (45.5)^b^	116.8 (66.3)^c^	<0.01	0.02
Vitamin D (µg)	3.6 (2.2)	2.8 (2.1)^a^	3.9 (2.4)^b^	3.8 (2.0)^b^	0.04	3.0 (2.0)	2.6 (1.6)	2.9 (2.1)	3.3 (2.1)	0.32	0.02
Omega-3 (g)	1.5 (1.2)	1.3 (0.9)^a^	1.6 (1.3)^b^	1.8 (1.3)^b^	0.16	1.2 (1.0)	1.0 (1.0)	1.2 (0.8)	1.3 (1.1)	0.25	0.01
Omega-6 (g)	6.5 (4.7)	6.5 (5.6)	6.2 (3.8)	6.9 (4.8)	0.44	5.5 (3.6)	4.6 (2.3)	5.8 (4.2)	5.8 (3.7)	0.62	<0.01

Superscript letters denote groups that are significantly different from one another.

* Kruskal-Wallis *H* Test.

** Paired sample t-test.

At month 3, average daily fibre (g) and folate intakes (µg) were significantly higher in those with a total PDQS score in the highest tertile compared with those in the lowest and middle tertile of total PDQS score (both *P* < 0.01). Average daily potassium intake (mg) was significantly higher in those who scored in the highest tertile of PDQS score compared with those in the lowest tertile (*P* = 0.01). Average daily vitamin C intake increased significantly across each tertile of PDQS score (*P* < 0.01).

Average daily nutritional intake from both food and dietary supplement sources was also analysed by tertile of total PDQS score (Supplementary Files 5). Similar trends were observed when including both foods and supplements as when considering foods-only data, with the exception that average intake of vitamin A (total retinol equivalents µg/d) was significantly higher in those that scored in the highest PDQS tertile compared with the lowest and middle tertile (*P* = 0.01) and there was no significant difference in average folate/folic acid intake between tertiles at month 3 when supplement data were also included.

Change in lifestyle data collected at month 3 were analysed to determine any changes in health or lifestyle over the 3-month study period and are presented in Supplementary Files (Table 6). ‘Only a small percentage of participants reported any changes (14.2% reported improvement in dietary intake; 19.2% reported increased physical activity; all other lifestyle changes reported were minimal (0.83–10.8%) at Month 3). As such, these data were too limited to perform further analysis with.’

A number of minor further amendments to the amended PDQS were made after the validation and reliability assessment based on feedback received from participants when completing the PDQS, and some individual food group results. Firstly, additional example foods were added to some food groups to clarify the diversity/breadth of particular food groups when making an estimate of frequency of consumption. Secondly, as the 21 PDQS food groups were presented individually to participants via the online questionnaire in the current study, the study team hypothesised that estimates may be improved by presenting related food groups together i.e. vegetable food groups (e.g. ‘other vegetables’, ‘cruciferous vegetables’, ‘dark green leafy vegetables’, ‘carrots’ and ‘legumes’) and also fruit groups (‘other fruits’, ‘whole citrus fruits’) and also ensuring ‘other vegetables’ and ‘other fruits’ groups are presented after the other specific fruit and vegetables category to assist with estimation of overall intake. The revised and final version is available in Supplementary Files Table 7.

## Discussion

This paper reports the amendment of the PDQS and its validity and reliability against a 4-day food diary in a UK/Irish population at risk of CVD. The original PDQS questionnaire was designed for a US population and, in order to adapt it for a UK/Irish population, food groups and descriptions were amended to reflect regional eating and cooking habits. Results indicate that the amended PDQS is a valid and reliable dietary screening tool for a UK/Irish population at risk of CVD.

Validity analyses indicated that total PDQS score from the PDQS was strongly positively correlated with total PDQS score obtained from FDs at both timepoints, with stronger correlations observed at month 3. In total, 76.2% of individual food group scores from the PDQS were strongly positively correlated with individual food scores derived from FDs at month 0. At month 3, 95.2% of the individual food scores from the PDQS were strongly positively correlated with individual food scores derived from food diaries with the exception of ‘legumes’. Intraclass correlation coefficients and weighted kappa both indicated a moderate level of agreement between measures for total PDQS, whilst Bland-Altman plots demonstrated a good level of validity between measures; providing further evidence of the validity of the PDQS. These results are similar to the level of agreement seen for energy and nutrient intake in validation studies that used food diaries as the reference measure^(12,23,24,32)^ and validations of different versions of the PDQS that used HEI-2015 derived from the Automated Self-Administered 24-hour (ASA24) Dietary Assessment Tool as a reference measure.^(13,14)^

The stronger validity correlations observed at month 3 compared with month 0 may be due to familiarity with the questionnaire and food diary completion process at the second administration, and therefore more accurate estimation of dietary intake. Stronger associations have also been observed at the second administration of the test and reference measures in an FFQ validity and reliability study in a childhood population that used a dietary record as a reference measure.^(33)^

In general, those who had a better diet quality, as determined via PDQS, had significantly higher intakes of micronutrients and fibre assessed via FD compared with those in the lowest tertile of PDQS score, which further supports the validity of the amended PDQS in terms of appropriately identifying those with suboptimal diet quality with a poorer intake of particular nutrients and micronutrients, and is a trend also observed in the validation of the rPDQS and the 24-hour and 30 d PDQS validation in US populations.^(13,14)^

Reliability analyses indicated that total PDQS score from the PDQS was strongly positively correlated with total PDQS score from PDQS at Month 3, with intraclass correlation coefficients and coefficient of variation indicating good reliability. The time interval between administration of the PDQS is similar to many reliability studies, which typically range between 1 and 12 months.^(17)^ Similar correlations were observed compared in other reliability studies, including the reliability of the rapid 13-item PDQS (rPDQS),^(14)^ the Diet Risk Score (DRS)^(29)^ and the nutrition component of the Rapid Eating and Activity Assessment for Patients (REAP) tool.^(31)^

Average total PDQS score derived from PDQS was significantly higher, indicating a better diet quality, at both month 0 and month 3, than average total PDQS score derived from FDs at month 0 and month 3. The difference in reference time frame may help to explain this observation, as PDQS referred to average intake over the previous 3 months, whilst the 4-day FDs captured a shorter 4-day period which may have been less representative of habitual intake. In the systematic review of dietary validation studies by Cade *et al.*,^(23)^ it was stated that, if FFQs are compared with weighed records, any lack of agreement can be attributed in part to within-subject variance that is inherent in the weighted record measure due to the shorter time frame and the more accurate record of dietary intake. Furthermore, a review of dietary validation and reliability studies^(23)^ discussed that individuals often have difficulty in estimating portion sizes of foods, both when examining displayed foods and when reporting about foods previously consumed and how if the individual cannot assign portion size, it can influence estimation of absolute nutrient intake^(23)^ So the ability to estimate portion size may have differed between the food diary where participants were asked to weigh or use portion size photos as guidance and the PDQS which was based on standard servings. Regardless of this, Cade *et al.* ^(23)^ recommend that weighed or dietary records are the preferred reference measure for FFQ validation rather than a 24-hour recall as the sources of error of a dietary record are less likely to be correlated with the error of the FFQ.^(23)^

In a systematic review of gender analysis in the development and validation of FFQs it was noted that, although gender can impact food choice and portions, gender differences are often not analysed during FFQ development and validation,^(34)^ and studies assessing the validity of the DRS and REAP scores did not present results by gender.^(29,31)^ Subgroup analyses were performed by gender in the present study despite the majority of the participants being female (78.3%), and agreement between measures was statistically significant in both females and males at both month 0 and 3; whilst stronger agreement was observed at month 0 in females compared with males and stronger agreement seen in males compared with females at month 3. In terms of reliability, stronger agreement was observed in females compared with males, but both were statistically significant. The consideration of gender is a notable aspect of the present study and indicates that the PDQS can be used as a diet screening tool for both males and females at risk of CVD.

As the vast majority of the sample (89.6%) were participants with overweight or obesity, it was not possible to perform subgroup analysis by BMI category (kg/m^2^). Data on changes to lifestyle were presented within Supplementary Files because only a small percentage of participants reported any improvement in dietary intake or physical activity at Month 3. As such, results were not presented in main paper as unlikely to have had an impact on PDQS validity and reliability assessment as both the test and reference measures were administered at both timepoints.

Strengths of the present study include the use of 4-day FDs as the reference measure. Seven-day FDs are considered to be one of the most robust ‘gold standard’ methods of dietary assessment available^(35,36)^ but 4-day FDs are widely considered a good practical alternative for many populations and study designs.^(37,38)^ Another strength of the current study is the sample size, which is larger than other validation studies^(24,26,27)^ and is at the high end of the sample size range recommended in a dietary validation study review by Cade *et al.*.^(23)^ Furthermore, this validation and reliability assessment was conducted in a well-defined population at risk of CVD on the island of Ireland and subgroup analyses was performed by gender. Furthermore, as the PDQS is designed to be a rapid diet quality screening tool, in that it is able to used in a time-sensitive clinical setting taking <10 minutes to complete (previously established as no more than 35 items),^(39)^ and comprised of food groups rather than specific food items, it is likely to be appropriate for use in more diverse populations than those studied, including regions other than UK and Ireland, and this is one of the strengths of its design and administration. In order to improve the validity and reliability of the PDQS further and informed by the results of this study, the authors recommend the following minor further amendments to the finalised PDQS (Supplementary Table 8) based on feedback received from participants when completing the PDQS, as described in Results section. These amendments are likely to improve the accuracy of the PDQS in determining diet quality by assisting participants with their estimations of intake as they better describe some food groups, give more extensive lists of example foods within some food groups to help participants categorise their intake correctly, and present related food groups together such as all vegetable groups and all fruit groups, to avoid errors is miscategorising some vegetables/fruits.

Limitations of the current study include the comparison of two self-reported methods of dietary assessment. It is well recognised that there is no objective and practical ‘gold standard’ method for directly assessing the validity of FFQs or dietary questionnaires^(23)^ and a dietary validation study can therefore only indicate whether both assessment methods obtain similar responses rather than accurate responses.^(23)^ However, as 7-day FDs are often considered to be one of the most robust and practical methods of dietary assessment, the choice of a 4-day FD was considered appropriate. Further to this, the method of administering the food diary over 4 consecutive days has been widely utilised in the literature^(40–42)^ although there is some evidence to suggest that administration on random days may obtain more representative data. As such, this may be a potential limitation but was considered appropriate and practical for the current study which was conducted during national lockdowns.^(43)^ In terms of reliability, the 3-month interval time frame chosen for the present study was intended to help account for any seasonal variations in dietary intake, but a short enough time frame to avoid capturing major changes in dietary habits that are more likely to occur years apart. Some literature suggests shorter intervals between assessments may make it more likely that latter responses are influenced by earlier responses, whilst too long an interval may reduce reproducibility as assessments are less able to detect true changes in dietary intake.^(23)^ It has been recommended that the time interval between repeat administrations of the test measures should minimise potential changes in dietary intake.^(23)^ In terms of sample population, the assessment of validity and reliability was conducted in a UK/Irish population at risk of CVD, but the majority of the sample recruited in the present sample were female (78.3%). No consistent differences in agreement were observed in the present study between genders so it is likely that this did not have a significant impact and would indicate generalisability to an adult population. The PDQS tool validated here has, however, not been designed for or validated in a population that follows an exclusively plant-based diet so it would be of interest to explore its application to more diverse population groups in future.

In conclusion, the amended PDQS demonstrated good validity and reproducibility in the current study and is appropriate for assessment of diet quality in a UK or Irish population at risk of CVD in place of more burdensome dietary assessment methods. The next step for this validated PDQS, or similar dietary scores, is to determine their association with NCD related-outcomes in European population cohorts, in a similar way as has been done with the variations of the PDQS in US cohorts,^(11,15–19)^ to further support is use in both the healthcare and research settings.

## Supporting information

Brennan et al. supplementary material 1 Brennan et al. supplementary material

## Supplementary material

For supplementary material accompanying this paper visit https://doi.org/10.1017/jns.2024.23. click here to view supplementary material
