# Supplementary material for: Validity and reproducibility of the Prime Diet Quality Score (PDQS) against a four-day food diary in adults at risk of cardiovascular disease on the island of Ireland
Source: J Nutr Sci. 2024 Aug 2;13:e29. doi: 10.1017/jns.2024.23 (PMC11704934; doi:10.1017/jns.2024.23)
Supplement: Brennan et al. supplementary material 1 — Brennan et al. supplementary material [file S2048679024000235sup001.docx]

**Supplementary Files**

**Table 1. PDQS Coding Guide for Composite/Ambiguous Food Diary Codes**

| **^Food item^** | **^PDQS code (food group)^** |
| --- | --- |
| ^Mashed potatoes (with/without milk/butter)^ | ^Code as ‘potatoes’ (food group 10)^ |
| ^Stir-fried vegetables^ | ^Code as per ‘dark green leafy vegetables’ (food group 11), cruciferous vegetables’ (food group 12), ‘carrots’ (food group 13), ‘other vegetables’ (food group 14) or ‘legumes’ (food group 15), where relevant.^ |
| ^Composite food codes^ | - ^Bolognese, meat pies, chilli con carne etc.: code as ‘red meat’ (food group 1) and apply a cap when calculating serving weights. Cap as per average portion calculated for relevant food group (Table 3).^ - ^Chicken stews, chicken pies, chicken curry etc: code as ‘poultry’ (food group 3). Cap as per average portion calculated for relevant food group (Table 2).^ - ^Lentil curries/stews: code as ‘legumes’ (food group 15). Cap as per average portion calculated for relevant food group (Table 2).^ - ^Fish pies/stews: code as ‘fish’ (food group 4). Cap as per average portion calculated for relevant food group (Table 2)^   ^Please note if portion of composite food consumed is small and less than that of capped weight, work out based on standard portion of composite food e.g. chicken casserole average serving is 260g and this food group is capped at 100g =38.5% so work out proportion based on amount of composite food consumed.^ |
| ^Dried fruit^ | ^Code as ‘other fruits’ (food group 17)^ |
| ^Spreads that are not butter^ | ^Uncoded (food group 22).^ |
| ^Fish/chicken, breaded, baked^ | ^Uncoded (food group 22)^ |
| ^Sausage rolls^ | ^Code as ‘processed meat’ (food group 2) but with a cap applied when calculating serving weights. Rather than cap as per food group category in a similar way to other composite codes described previously, cap more specifically as 40g sausage contribution per 1 sausage roll^ |
| ^Biscuits/confectionery^ | ^Biscuits or half-coated chocolate biscuits code as ‘sweet baked foods’ (food group 9).^  ^Fully coated types such as KitKat should be coded as ‘desserts, puddings, confectionery’ (food group 20).^ |
| ^Sugar^ | ^Uncoded (food group 22).^ |
| ^Jam^ | ^Uncoded (food group 22)^ |
| ^Macaroni cheese^ | ^Uncoded (food group 22)^ |
| ^Peanut butter^ | ^Uncoded (food group 22).^ |
| ^Soup^ | ^Code homemade vegetable soup as ‘other vegetables’ (food group 14). All canned soup uncoded.^ |
| ^Prawns or shellfish^ | ^Code as ‘fish’ (food group 4) if not battered/crumbed/coated.^ |
| ^Duck, roasted, meat only^ | ^Uncoded (food group 22).^ |
| ^Hard/soft cheese^ | ^Only hard cheeses care coded within ‘whole milk dairy’ (food group 6) including: Cheddar, Red Leicester, Wenselydale, Percorino, Parmesan, Grana Padano, Gouda^ |
| ^Black pudding^ | ^Code as ‘processed meat’ (food group 2).^ |
| ^Omelette^ | ^Code as ‘whole eggs’ (food group 5).^ |
| ^Olives^ | ^Uncoded (food group 22).^ |
| ^Tomato passata/canned^ | ^Code as ‘other vegetables’ (food group 14).^ |
| ^Fruit smoothie^ | ^Code as ‘other fruits’ (food group 17).^ |
| ^Cereal bars with and without chocolate^ | ^Uncoded (food group 22).^ |
| ^Garlic^ | ^Uncoded (food group 22)^ |
| ^Onion rings/oven chips/potato waffles/smilies^ | ^Coded as 7 typically, but can check context e.g. onion rings, frozen, from food diary if in doubt^ |
| ^Pancakes/crumpets^ | ^Uncoded (food group 22).^ |
| ^Wholegrains^ | ^Code 8 should be used to code this food group category to include the following food types: wholegrain breads (wholewheat, wholemeal and wholegrain breads ONLY),wholegrain breakfast cereals (porridge, Weetabix, Shredded Wheat ONLY), brown rice, wholewheat pasta, Bran Flakes, All-Bran, brown bread, brown soda bread, wheaten bread, 50/50 bread, oatcakes, Fruit and Fibre.^ |

**Table 2. PDQS Food Category Average Serving Sizes**

| **PDQS Food Category** | | **Serving weights of each example component within food group** |
| --- | --- | --- |
| 1 | **Red Meat e.g.**  minced beef  (lasagne, bolognaise, Cottage Pie, beef stew/casserole),**  steak,  pork chop,  lamb. | **140g**  ***note composite dishes capped as detailed in text**  **144g**  **75-170g** depending on cut (average: **122.5g)**  **90g** (roast) Chops: **50-120g**: average: **85g** |
| **Average serving weight** | | **116.3g** |
| 2 | **Processed Meats e.g.**  sausages,  bacon,  ham,  corned beef,  tinned meat. | **80g (2)**  **50g (2)**  **46g** (2 slices)  1 thin slice **28g**/ thick **50g**  Small can **198g** |
| **Average serving weight** | | **75.3g** |
| 3 | **Poultry e.g.**  chicken or turkey breast/fillet,  slices (no batter/crumbs) | **130g**  **70g** (small average. portion e.g. sandwiches) |
| **Average serving weight** | | **100g** |
| 4 | **Fish e.g.**  cod,  haddock,  salmon,  tuna,  mackerel (no batter/crumbs) | **120g**  **120g**  **100g**  **45**g sandwich/**92g** salad  **160g** grilled/can **125g** |
| **Average serving weight** | | **108.8g** |
| 5 | **Whole Eggs e.g.**  boiled,  scrambled,  poached (not fried) | **50g (1) 100g (2)**  **120g (2)**  **100g (2)** |
| **Average serving weight** | | **92.5g** |
| 6 | **Whole Milk Dairy Foods e.g.**  whole/fullfat milk,  hard cheese,  butter,  full-fat yoghurts | **100ml** (cereal) or **200ml** average glass  **30g** matchbox size **45**g average in sandwich  Medium spread bread 1 slice **10g**  **125**g |
| **Average serving weight** | | **85g** |
| 7 | High-Fat Foods e.g.  fast food takeaways  (chips, fried chicken/fish/burgers),  fried breakfasts  (e.g. fried breads,eggs),  roast potatoes,  crisps | **165g, 70g, 170g, 36g** without bun  **31**g, **60**g (1 fried egg)  **200g** ave portion/ **85g** (1 ave roast)  **25g** |
| **Average serving weight** | | **93.5g** |
| 8 | Whole-Grain Foods e.g.  wholegrain breads,  wholegrain breakfast cereals  (porridge, Weetabix, Shredded Wheat),  brown pasta  brown/rice. | **72g (2)**  **35g, 40g (2), 45g (2)**  **230g**  **180g** |
| **Average serving weight** | | **100.3g** |
| 9 | **Sweet Baked Foods e.g.**  cakes,  buns,  muffins,  cookies,  scones | **60g**  **28g**  **85g**  **17g**  **48g** |
| **Average serving weight** | | **47.5g** |
| 10 | **Potatoes e.g.**  boiled,  baked,  mashed (not chips, roast) | **175g** average portion  **160g**  **180g** (BNF portion guide)-FSA book only has wt for one scoop |
| **Average serving weight** | | **171.7g** |
| 11 | Dark Green Leafy Vegetables e.g.  spinach,  lettuce,  kale,  spring greens (includes frozen, tinned) | **90g**  **30g**  Not available  **95g** |
| **Average serving weight** | | **71.6g** |
| 12 | Cruciferous Vegetables e.g. broccoli,  cauliflower,  cabbage,  brussels sprouts (includes frozen, tinned) | **85g**  **90g**  **95g**  **90g** |
| **Average serving weight** | | **90g** |
| 13 | Carrots e.g.  raw, boiled, steamed, mashed, microwaved, frozen, tinned | **60g** |
| **Average serving weight** | | **60g** |
| 14 | **Other vegetables e.g.**  mushrooms,  corn,  turnip,  cucumber,  tomatoes (includes frozen, tinned) | **56g**  **85g**  **60g**  **23g**  **85g** (1 medium) |
| **Average serving weight** | | **61.8** |
| 15 | Legumes e.g.  peas,  baked beans,  kidney beans,  lentils,  chickpeas (includes frozen, tinned) | **70g**  **135g**  **120g** (BNF)  **120g** (BNF)  **90g** |
| **Average serving weight** | | **107g** |
| 16 | **Whole Citrus Fruit**  e.g. oranges,  grapefruit,  lemons (not fruit juices) | **160g**  **340g (not included in average)**  Just as a 20g slice/juice for drinks |
| **Average serving weight** | | **90g** |
| 17 | Other fruits e.g.  apples,  pears,  bananas,  strawberries,  raspberries,  grapes,  melon (includes frozen, tinned) | **100g**  **150g**  **100g**  **100g**  **60g**  **100g** (small bunch)  **200g** (slice without skin) |
| **Average serving weight** | | **115.7g** |
| 18 | Liquid Vegetable Oils  e.g. olive oil,  rapeseed oil (not palm oil or coconut oil) | **11g** (1 tbsp)  **11g** (1 tbsp) |
| **Average serving weight** | | **11g** |
| 19 | Nuts e.g.  almonds,  peanuts,  cashew,  hazelnuts,  brazil nuts (unsalted only) | **13g** (6 whole)  **13g** (10 whole)  **10g** (10 whole)  -  **10g** (3 whole) |
| **Average serving weight** | | **11.5g** |
| 20 | **Desserts, Puddings & Confectionery e.g.** ice cream,  custard,  sponge puddings,  crème brulee,  fruit pies,  cheesecakes,  chocolate,  sweets | **75g**  **120g**  **110g**  **100g**  **110g**  **120g**  **54g**  **30g** (tube**)** |
| **Average serving weight** | | **89.9** |
| 21 | **Sugar Sweetened Beverages e.g.**  cola,  sodas,  lemonade,  flavoured juices,  energy drinks (not diet/sugar-free varieties) | **200**  **200**  **200**  **200**  **200** |
| **Average serving weight** | | **261.4g** |

**Table 3. Spearman correlation coefficients (r), weighted kappa (K) (where appropriate) and ICCs for PDQS questionnaire and PDQS scores derived from 4-day food diaries at Month 0 and Month 3; and Spearman correlation coefficients, ICCs and coefficient of variation (where appropriate) for PDQS questionnaire at Month 0 and Month 3 in FEMALES**

| **PDQS Food Groupings** | **Month 0 (n=84)** | | **Month 3 (n=81)** | | **Reliability (n=86)** | | |
| --- | --- | --- | --- | --- | --- | --- | --- |
|  | **Validity (r)**  **K(SE)** | **ICC (95% CI)** | **Validity (r)**  **K (SE)** | **ICC (95% CI)** | **r**  **K (SE)**  **(n=86)** | **ICC (95% CI)** | **Coeff. of variation** |
| Total PDQS Score  Red Meat | 0.60**  0.41 (0.08) | 0.69 (0.40-0.83) | 0.59**  0.37 (0.08) | 0.67 (0.36-0.82) | 0.79**  0.68 (0.06) | 0.88 (0.82-0.92) | 0.104 |
|  |  |  |  |  |  |  |  |
|  | 0.28** | 0.44 (0.14-0.64) | 0.30** | 0.49 (0.21-0.67) | 0.68** | 0.77 (0.67-0.86) |  |
| Processed Meat | 0.22* | 0.39 (0.07-0.60) | 0.25* | 0.35 (0.01-0.58) | 0.54** | 0.65 (0.47-0.77) |  |
| Poultry | 0.31** | 0.41 (0.09-0.62) | 0.36** | 0.49 (0.22-0.67) | 0.56** | 0.70 (0.54-0.81) |  |
| Fish | 0.29** | 0.38 (0.06-0.60) | 0.41** | 0.53 (0.28-0.70) | 0.67** | 0.80 (0.69-0.87) |  |
| Whole Eggs | 0.31** | 0.51 (0.26-0.68) | 0.46** | 0.67 (0.49-0.79) | 0.66** | 0.80 (0.69-0.87) |  |
| Whole Milk Dairy Foods | 0.08 | 0.15 (-0.270-0.43) | 0.33** | 0.49 (0.22-0.67) | 0.38** | 0.59 (0.38-0.74) |  |
| High-Fat Foods | 0.10 | 0.12 (-0.17-0.36) | 0.17 | 0.19 (-0.13-0.44) | 0.26* | 0.53 (0.28-0.70) |  |
| Wholegrain Foods | 0.47** | 0.66 (0.47-0.78) | 0.47** | 0.01 (-0.02-0.06) | 0.55** | 0.72 (0.57-0.82) |  |
| Sweet Baked Foods | 0.34** | 0.47 (0.20-0.66) | 0.26* | 0.40 (0.09-0.61) | 0.47** | 0.66 (0.47-0.77) |  |
| Potatoes | 0.20 | 0.31 (-0.03-0.54) | 0.42** | 0.57 (0.33-0.72) | 0.62** | 0.77 (0.64-0.85) |  |
| Dark Green Leafy Vegetables | 0.19 | 0.20 (-0.13-0.45) | 0.45** | 0.46 (0.02-0.69) | 0.58** | 0.73 (0.59-0.83) |  |
| Cruciferous Vegetables | 0.29** | 0.44 (0.140-0.64) | 0.16 | 0.27 (-0.09-0.51) | 0.61** | 0.75 (0.62-0.84) |  |
| Carrots | 0.25* | 0.36 (0.04-0.58) | 0.34** | 0.53 (0.27-0.69) | 0.61** | 0.76 (0.64-0.85) |  |
| Other Vegetables | 0.31* | 0.36 (-0.01-0.60) | 0.33** | 0.40 (-0.02--0.64) | 0.56** | 0.73 (0.58-0.82) |  |
| Legumes | 0.35** | 0.51 (0.24-0.68) | 0.08 | 0.11 (-0.39-0.43) | 0.61** | 0.76 (0.63-0.84) |  |
| Whole Citrus Fruit | 0.63** | 0.74 (0.60-0.83) | 0.53** | 0.65 (0.44-0.78) | 0.67** | 0.79 (0.68-0.86) |  |
| Other Fruits | 0.49** | 0.71 (0.55-0.81) | 0.51** | 0.70 (0.54-0.81) | 0.61** | 0.79 (0.68-0.86) |  |
| Liquid Vegetable Oils  Nuts | 0.22*  0.40** | 0.26 (-0.08-0.51)  0.61 (0.39-0.75) | 0.29**  0.50** | 0.35 (-0.03-0.59)  0.71 (0.54-0.81) | 0.71**  0.73** | 0.83 (0.73-0.87)  0.85 (0.77-0.90) |  |
| Desserts, Pudding, Confectionery | 0.10 | 0.20 (-0.21-0.48) | 0.22 | 0.37 (0.02-0.59) | 0.48** | 0.62 (0.43-0.75) |  |
| Sugar Sweetened Beverages | 0.39** | 0.55 (0.30-0.71) | 0.35** | 0.58 (0.35-0.73) | 0.70** | 0.81 (0.71-0.880) |  |

*R=Spearman correlation coefficient; *significant at the 0.05 level (2-tailed); **significant at the 0.01 level (2-tailed); K=weighted kappa statistics, SE: standard error; ICC: intraclass correlation coefficient.*

| **PDQS Food Groupings** | **Month 0 (n=24)** | | **Month 3 (n=24)** | | **Reliability (n=24)** | | |
| --- | --- | --- | --- | --- | --- | --- | --- |
|  | **Validity (r)**  **K(SE)** | **ICC (95% CI)** | **Validity (r)**  **K (SE)** | **ICC (95% CI)** | **r**  **K (SE)**  **(n=86)** | **ICC (95% CI)**  **N=24)** | **Coeff. of variation** |
| Total PDQS Score  Red Meat | 0.51**  0.38 (0.16) | 0.70 (0.32-0.87) | 0.78**  0.52 (0.14) | 0.82 (0.27-0.94) | 0.71** 0.65 (0.12) | 0.85 (0.64-0.93) | 0.131 |
|  |  |  |  |  |  |  |  |
|  | 0.68** | 0.79 (0.52-0.91) | 0.47* | 0.65 (0.19-0.85) | 0.67** | 0.81 (0.57-0.92) |  |
| Processed Meat | 0.40 | 0.53 (-0.09-0.80) | 0.06 | 0.13 (-1.00-0.63) | 0.61** | 0.79(0.53-0.91) |  |
| Poultry | 0.38 | 0.55 (-0.03-0.81) | 0.34 | 0.48 (-0.23-0.78) | 0.53** | 0.72 (0.34-0.88) |  |
| Fish | 0.08 | 0.26 (-0.65-0.67 | 0.51* | 0.58 (0.10-0.82) | 0.78** | 0.88 (0.72-0.95) |  |
| Whole Eggs | 0.090 | 0.07 (-1.25-0.61) | 0.64** | 0.75 (0.39-0.89) | 0.51* | 0.68 (0.25-0.86) |  |
| Whole Milk Dairy Foods | 0.32 | 0.52 (-0.05-0.80) | 0.44* | 0.61 (0.13-0.83) | 0.55** | 0.73 (0.37-0.88) |  |
| High-Fat Foods | 0.36 | 0.34 (-0.23-0.68) | 0.31 | 0.24 (-0.23-0.60) | 0.07 | 0.00 (-1.41-0.58) |  |
| Wholegrain Foods | 0.62** | 0.79 (0.50-0.91) | 0.67** | 0.05 (-0.06-0.23) | 0.80** | 0.91 (0.78-0.96) |  |
| Sweet Baked Foods | 0.33 | 0.48 (-0.18-0.77) | 0.49* | 0.66 (0.22-0.85) | 0.48* | 0.64 (0.18-0.84) |  |
| Potatoes | 0.67** | 0.79 (0.51-0.91) | 0.71** | 0.79 (0.51-0.91) | 0.71** | 0.82 (0.58-0.92) |  |
| Dark Green Leafy Vegetables | 0.08 | 0.07 (-0.68- 0.55) | 0.45* | 0.38 (-0.22-0.71) | 0.74** | 0.85 (0.660-0.94) |  |
| Cruciferous Vegetables | 0.34 | 0.51 (-0.13-0.79) | 0.19 | 0.38 (-0.27-0.71) | 0.72** | 0.83 (0.62-0.93) |  |
| Carrots | 0.44* | 0.51 (-0.15-0.79) | 0.30 | 0.45 (-0.26-0.76) | 0.74** | 0.80 (0.52-0.91) |  |
| Other Vegetables | 0.53** | 0.61 (0.04-0.84) | 0.52** | 0.66 (0.25-0.85) | 0.67** | 0.81 (0.550-0.92) |  |
| Legumes | 0.42* | 0.54 (-0.02-0.80) | 0.32 | 0.42 (-0.25-0.74) | 0.34 | 0.53 (-0.12-0.80) |  |
| Whole Citrus Fruit | 0.69** | 0.80 (0.55-0.92) | 0.48* | 0.59 (0.09-0.82) | 0.83** | 0.91 (0.79-0.96) |  |
| Other Fruits | 0.84** | 0.91 (0.80-0.96) | 0.80** | 0.89 (0.75-0.95) | 0.83** | 0.91 (0.79-0.96) |  |
| Liquid Vegetable Oils  Nuts | -0.08 - | -0.18 (-0.61-0.38)  0.000 (-1.11-0.55) | -0.25 - | -0.14 (-0.66-0.34)  0.00 (-1.11-0.55) | 0.48* 0.30 | 0.72 (0.34-0.88)  0.78 (0.48-0.90) |  |
| Desserts, Pudding, Confectionery | 0.05 | 0.11 (-1.18-0.62) | 0.34 | 0.53 (-0.12-0.80) | 0.33 | 0.49 (-0.14-0.77) |  |
| Sugar Sweetened Beverages | 0.31 | 0.47 (-0.24-0.77) | 0.47* | 0.82 (0.58-0.92) | 0.56** | 0.66 (0.21-0.86) |  |

**Table 4. Spearman correlation coefficients (r), weighted kappa (K) (where appropriate) and ICCs for PDQS questionnaire and PDQS scores derived from 4-day food diaries at Month 0 and Month 3; and Spearman correlation coefficients, ICCs and coefficient of variation (where appropriate) for PDQS questionnaire at Month 0 and Month 3 in MALES**

*R=Spearman correlation coefficient; *significant at the 0.05 level (2-tailed); **significant at the 0.01 level (2-tailed); K=weighted kappa statistics, SE: standard error; ICC: intraclass correlation coefficient.*

**Table 5. Average daily nutrient intake by tertile of PDQS total score from amended PDQS (food and supplements)**

| **Nutrient intake** | **Average daily intake**  **Mean (SD)** | **Month 0** | | | | **Average daily intake**  **Mean (SD)** | **Month 3** | | | | **Difference in nutrient intake Month 0-Month3**  **P** |
| --- | --- | --- | --- | --- | --- | --- | --- | --- | --- | --- | --- |
|  |  | **PDQS Total Score** | | | |  | **PDQS Total Score** | | | |  |
|  |  | **PDQS Total Score**  **Tertile 1 (n=36)**  **Mean (SD)** | **PDQS Total Score**  **Tertile 2 (n=38)**  **Mean (SD)** | **PDQS Total Score**  **Tertile 3 (n=41)**  **Mean (SD)** | **Difference between tertiles**  **P*** |  | **PDQS Total Score**  **Tertile 1 (n=31)**  **Mean (SD)** | **PDQS Total Score**  **Tertile 2 (n=40)**  **Mean (SD)** | **PDQS Total Score**  **Tertile 3 (n=39)**  **Mean (SD)** | **Difference between tertiles**  **P*** |  |
| Energy (kcal) | 1949.5 (492.4) | 1945.15 (556.29) | 1988.30 (540.04) | 1915.40 (383.35) | 0.95 | 1794.4 (494.6) | 1888.70 (602.70) | 1763.11 (458.83) | 1761.17 (450.36) | 0.94 | <0.01 |
| Carbohydrate (g) | 205.1 (52.6) | 207.65 (50.73) | 210.03 (62.45) | 198.18 (43.70) | 0.56 | 189.3 (61.2) | 201.28 (81.69) | 184.91 (57.61) | 185.42 (49.67) | 0.76 | <0.01  0.02 |
| Protein (g) | 80.9 (23.7) | 77.69 (29.97) | 84.02 (21.08) | 80.61 (19.76) | 0.23 | 76.6 (19.8) | 77.52 (23.22) | 74.66 (17.84) | 78.00 (19.63) | 0.94 |  |
| Total Fat (g) | 81.0 (26.4) | 79.80 (26.12) | 82.86 (29.53) | 80.07 (23.93) | 0.94 | 74.2 (24.8) | 79.42 (24.11) | 72.10 (22.77) | 72.74 (27.36) | 0.39 | <0.01 |
| Saturated Fat (g) | 29.5 (11.4) | 30.40 (11.69) | 31.54 (13.58) | 26.71 (7.97) | 0.17 | 27.9 (10.4) | 31.67 (10.85) | 27.35 (8.03) | 25.85 (11.60) | 0.06 | 0.19 |
| Free Sugars (g) | 40.0 (26.1) | 44.54 (28.56) | 41.60 (30.26) | 34.54 (18.05) | 0.34 | 35.8 (23.8) | 44.79 (32.36) | 34.43 (22.42) | 31.00 (15.72) | 0.34 | 0.13 |
| Alcohol (g) | 25.3 (17.6) | 23.92 (17.37) | 25.87 (19.06) | 25.84 (17.39) | 0.95 | 20.9 (17.0) | 19.85 (17.48) | 21.74 (19.97) | 20.56 (13.79) | 0.84 | 0.15 |
| NSP Englyst Fibre (g) | 15.3 (5.1) | 12.73 (3.99)^a^ | 14.99 (4.88)^a^ | 17.75 (4.96)^b^ | <0.01 | 14.4 (5.4) | 11.64 (5.02)^a^ | 13.59 (4.30)^a^ | 17.12 (5.44)^b^ | <0.01 | 0.03 |
| Fibre (g) | 20.8 (6.7) | 17.43 (5.56^)a^ | 20.37 (5.72)^b^ | 24.25 (7.00)^c^ | <0.01 | 19.0 (6.6) | 15.45 (5.84)^a^ | 18.19 (5.48)^b^ | 22.37 (6.59)^c^ | <0.01 | <0.01 |
| Iron (mg) | 15.3 (30.9) | 10.99 (4.40) | 19.14 (50.17) | 15.16 (16.33) | 0.08 | 15.2 (30.8) | 10.53 (4.25) | 18.11 (47.59) | 15.36 (16.28) | 0.06 | 0.73 |
| Sodium (mg) | 2115.6 (687.5) | 2139.22 (623.99) | 2220.87 (805.31) | 1992.47 (610.01) | 0.41 | 2078.4 (758.7) | 2210.15 (822.05) | 2037.39 (676.85) | 2029.18 (800.85) | 0.57 | 0.64 |
| Potassium (mg) | 3245.3 (832.2) | 2921.79 (924.55)^a^ | 3252.88 (720.75)^a,b^ | 3518.71 (766.88)^b^ | <0.01 | 3120.3 (825.1) | 2831.12 (799.93)^a^ | 3024.46 (727.09)^a,b^ | 3418.88 (860.44)^b^ | 0.01 | 0.01 |
| Calcium (mg) | 871.2 (347.8) | 882.45 (502.80) | 864.02 (270.40) | 868.36 (234.47) | 0.65 | 860.4 (379.8) | 856.89 (301.29) | 820.25 (356.80) | 903.94 (449.73) | 0.63 | 0.74 |
| Iodine (ug) | 145.7 (61.7) | 116.14 (35.46)^a^ | 150.66 (64.57)^b^ | 166.64 (68.02)^b^ | <0.01 | 148.7 (80.1) | 129.82 (67.55) | 142.46 (69.71) | 168.13 (94.44) | 0.08 | 0.85 |
| Thiamin (mg) | 1.8 (0.8) | 1.73 (0.95) | 1.73 (0.58) | 1.93 (0.75) | 0.21 | 1.8 (1.2) | 1.54 (0.65) | 1.62 (0.72) | 2.07 (1.81) | 0.16 | 0.75 |
| Riboflavin (mg) | 2.0 (0.8) | 1.89 (1.01) | 1.87 (0.65) | 2.10 (0.83) | 0.17 | 1.9 (0.9) | 1.72 (0.66) | 1.78 (0.85) | 2.07 (1.24) | 0.37 | 0.30 |
| Niacin (mg) | 38.6 (13.1) | 39.36 (16.29) | 38.71 (11.44) | 37.79 (11.46) | 0.97 | 36.9 (13.2) | 22.57 (13.55) | 20.60 (8.00) | 20.66 (9.01) | 0.99 | 0.11 |
| Folate (natural and folic acid supplements) | 454.0 (755.09) | 638.63  1244.11 | 321.55  105.43 | 372.01  144.43 | 0.66 | 868.61 (1570.0) | 1269.33  (2711.39) | 647.59  (308.55) | 683.43  (311.77) | 0.61 | 0.74 |
| Vitamin A (Total Retinol Equivalents) (ug) | 882.6 (608.2) | 711.78 (402.68) | 896.25 (565.38) | 1017.61 (757.44) | 0.29 | 914.6 (547.9) | 714.25 (295.06)^a^ | 874.49 (512.53)^a^ | 1094.53 (661.06)^b^ | 0.01 | 0.71 |
| Vitamin B12 (Cobalamin) (µg) | 9.1 (25.6) | 8.55 (17.46) | 6.04 (2.86) | 12.41 (40.49) | 0.31 | 8.6 (25.9) | 5.48 (4.05) | 7.48 (15.83) | 11.9 (39.58) | 0.48 | <0.05 |
| Vitamin C (mg) | 148.1 (204.3) | 141.60 (257.32)^a^ | 129.96 (176.45)^b^ | 171.43 (179.38)^c^ | <0.01 | 146.6 (257.9) | 73.61 (98.02)^a^ | 139.29 (307.84)^a^ | 204.58 (269.48)^b^ | <0.01 | 0.88 |
| Vitamin D (µg) | 11.5 (25.8) | 6.85 (14.17)^a^ | 13.27 (38.48)^a,b^ | 13.84 (17.01)^b^ | 0.02 | 13.9 (26.4) | 24.58 (46.54) | 8.27 (11.09) | 12.37 (14.39) | 0.12 | 0.23 |
| Omega-3 (g) | 1.8 (2.3) | 1.26 (0.89) | 1.93 (2.46) | 2.21 (2.78) | 0.12 | 1.4 (1.6) | 1.11 (1.01) | 1.27 (0.89) | 1.67 (2.36) | 0.45 | 0.38 |
| Omega-6 (g) | 6.7 (4.8) | 6.50 (5.56) | 6.37 (3.82) | 7.09 (4.93) | 0.41 | 5.5 (3.5) | 4.68 (2.22) | 5.80 (4.22) | - 1. (3.45) | 0.53 | <0.01 |

***Kruskal-Wallis H Test; superscript letters denote groups that are significantly different from one another.**

**Table 6.**

| **Improvement or deterioration in health or lifestyle** | **Reported Change in Lifestyle Behaviours during the study period** | **N (%)** |
| --- | --- | --- |
| Participants reporting perceived improvement in health and lifestyle behaviours over the study period | Participants reporting perceived improvement in dietary intake during the study period | 17 (14.2) |
|  | Participants reporting perceived weight loss during the study period | 1 (0.83) |
|  | Participants reporting drinking less during study period | 9 (7.5) |
|  | Participants reporting smoking less during study period | 1 (0.83) |
|  | Participants reporting increased physical activity during the study period | 23 (19.2) |
| Participants reporting perceived deterioration in health and lifestyle behaviours over the study period | Participants reporting perceived deterioration in dietary intake during the study period | 3 (2.5) |
|  | Participants reporting perceived weight gain during the study period | 1 (0.83) |
|  | Participants reporting drinking and smoking more during the study period | 2 (1.7) |
|  | Participants reporting reduced physical activity during study period | 13 (10.8) |
|  | Participants reporting a significant deterioration to their health during the study period | 11(9.2) |
|  | Participants reporting change in medication during the study period | 9 (7.5) |

**Table 7. Amended PDQS Following Validation and Reliability Results.**

| **Prime Diet Quality Score** | | | | | | |
| --- | --- | --- | --- | --- | --- | --- |
| For each question, mark the column indicating how often **on average** you have used the item(s) **during the past 3 months** | | Less than once per week | Once per week | 2-4 per week | Nearly daily or daily | Twice or more per day |
| 1 | Red Meat e.g. minced beef (lasagne, bolognaise, Cottage Pie, Irish Stew), beef stew/casserole, steak, pork chop, lamb. |  |  |  |  |  |
| 2 | Processed Meats e.g. sausages, bacon, ham, corned beef, tinned meat, chorizo, pepperoni. |  |  |  |  |  |
| 3 | Poultry e.g. chicken or turkey breast/fillet, slices (no batter/crumbs), chicken curry/casseroles. |  |  |  |  |  |
| 4 | Fish e.g. cod, haddock, salmon, tuna, mackerel, trout, hake, sardines, fish curries/stews (no batter/crumbs) |  |  |  |  |  |
| 5 | Whole Eggs e.g. boiled, scrambled, poached (not fried) |  |  |  |  |  |
| 6 | Whole Milk Dairy Foods e.g. whole/full-fat milk, hard cheese (e.g. cheddar, Red Leicester), butter, full-fat yoghurts |  |  |  |  |  |
| 7 | High-Fat Foods e.g. fast food takeaways (chips, fried chicken/fish/burgers), fried breakfasts (e.g. fried breads, eggs), roast potatoes, crisps |  |  |  |  |  |
| 8 | Whole-Grain Foods e.g. wholegrain breads, wholegrain breakfast cereals (porridge, Weetabix, Shredded Wheat All-Bran, Bran Flakes), brown pasta/rice. |  |  |  |  |  |
| 9 | Sweet Baked Foods e.g. cakes, buns, muffins, cookies, scones |  |  |  |  |  |
| 10 | Potatoes e.g. boiled, baked, mashed (not chips, roast) |  |  |  |  |  |
| 11 | Dark Green Leafy Vegetables e.g. spinach, lettuce, kale, spring greens (includes frozen, tinned) |  |  |  |  |  |
| 12 | Cruciferous Vegetables e.g. broccoli, cauliflower, cabbage, brussels sprouts (includes frozen, tinned) |  |  |  |  |  |
| 13 | Carrots e.g. raw, boiled, steamed, mashed, microwaved, frozen, tinned |  |  |  |  |  |
| 14 | Other vegetables e.g. mushrooms, corn, turnip, cucumber, tomatoes, onions, peppers, leeks, homemade vegetable soup (includes frozen, tinned vegetables) |  |  |  |  |  |
| 15 | Legumes e.g. peas, baked beans, kidney beans, lentils, chickpeas (includes frozen, tinned) |  |  |  |  |  |
| 16 | Whole Citrus Fruit e.g. oranges, grapefruit, lemons (not fruit juices) |  |  |  |  |  |
| 17 | Other fruits e.g. apples, pears, bananas, strawberries, raspberries, grapes, melon, blueberries, plums (includes frozen, tinned) |  |  |  |  |  |
| 18 | Liquid Vegetable Oils e.g. olive oil, rapeseed oil (not palm oil or coconut oil) |  |  |  |  |  |
| 19 | Nuts e.g. almonds, peanuts, cashew, hazelnuts, brazil nuts (unsalted only) |  |  |  |  |  |
| 20 | Desserts, Puddings and Confectionery e.g. ice cream, ice lollies, custard, sponge puddings, crème brulee, fruit pies, cheesecakes, chocolate, sweets |  |  |  |  |  |
| 21 | Sugar Sweetened Beverages e.g. cola, sodas, lemonade, flavoured juices, energy drinks (not diet/sugar-free varieties) |  |  |  |  |  |
